# Nanotechnology approaches to crossing the blood-brain barrier and drug delivery to the CNS

**DOI:** 10.1186/1471-2202-9-S3-S4

**Published:** 2008-12-10

**Authors:** Gabriel A Silva

**Affiliations:** 1 Departments of Bioengineering and Ophthalmology, and Neurosciences Program, University of California, San Diego, 9415 Campus Point Drive, La Jolla, California 92037-0946, USA

## Abstract

Nanotechnologies are materials and devices that have a functional organization in at least one dimension on the nanometer (one billionth of a meter) scale, ranging from a few to about 100 nanometers. Nanoengineered materials and devices aimed at biologic applications and medicine in general, and neuroscience in particular, are designed fundamentally to interface and interact with cells and their tissues at the molecular level.  One particularly important area of nanotechnology application to the central nervous system (CNS) is the development of technologies and approaches for delivering drugs and other small molecules such as genes, oligonucleotides, and contrast agents across the blood brain barrier (BBB).  The BBB protects and isolates CNS structures (i.e. the brain and spinal cord) from the rest of the body, and creates a unique biochemical and immunological environment.  Clinically, there are a number of scenarios where drugs or other small molecules need to gain access to the CNS following systemic administration, which necessitates being able to cross the BBB.  Nanotechnologies can potentially be designed to carry out multiple specific functions at once or in a predefined sequence, an important requirement for the clinically successful delivery and use of drugs and other molecules to the CNS, and as such have a unique advantage over other complimentary technologies and methods.  This brief review introduces emerging work in this area and summarizes a number of example applications to CNS cancers, gene therapy, and analgesia.

## Introduction

Nanotechnologies are materials and devices that have a functional organization in at least one dimension on the nanometer (one billionth of a meter) scale, ranging from a few to about 100 nanometers. Nanoengineered materials and devices aimed at biologic applications and medicine in general, and neuroscience in particular, are designed fundamentally to interface and interact with cells and their tissues at the molecular level. The potential of nanotechnological applications to biology and medicine arise from the fact that they exhibit bulk mesoscale and macroscale chemical and/or physical properties that are unique to the engineered material or device and not necessarily possessed by the molecules alone. This supports the development of nanotechnologies that can potentially carry out multiple specific functions at once or in a predefined sequence, which is an important property for the clinically successful delivery of drugs and other molecules to the central nervous system (CNS).

An ability to cross the blood-brain barrier (BBB) to deliver drugs or other molecules (for example, oligonucleotides, genes, or contrast agents) while potentially targeting a specific group of cells (for instance, a tumor) requires a number of things to happen together. Ideally, a nanodelivery-drug complex would be administered systemically (for example, intravenously) but would find the CNS while producing minimal systemic effects, be able to cross the BBB and correctly target cells in the CNS, and then carry out its primary active function, such as releasing a drug. These technically demanding obstacles and challenges will require multidisciplinary solutions between different fields, including engineering, chemistry, cell biology, physiology, pharmacology, and medicine. Successfully doing so will greatly benefit the patient. Although this ideal scenario has not yet been realized, a considerable body of work has been done to develop nanotechnological delivery strategies for crossing the BBB.

## Applications to drugs and other molecules

A significant amount of work using nanotechnological approaches to crossing the BBB has focused on the delivery of antineoplastic drugs to CNS tumors. For example, radiolabeled polyethylene glycol (PEG)-coated hexadecylcyanoarcylate nanospheres have been tested for their ability to target and accumulate in a rat model of gliosarcoma [1]. Another group has encapsulated the antineoplasitc drug paclitaxel in polylactic co-glycolic acid nanoparticles, with impressive results. *In vitro *experiments with 29 different cancer cell lines (including both neural and non-neural cell lines) demonstrated targeted cytotoxicity 13 times greater than with drug alone [2]. Using a variety of physical and chemical characterization methods, including different forms of spectroscopy and atomic force microscopy, the investigators showed that the drug was taken up by the nanoparticles with very high encapsulation efficiencies and that the release kinetics could be carefully controlled. Research focusing on the delivery of many of the commonly used antineoplastic drugs is important because most of these drugs have poor solubility under physiologic conditions and require less than optimal vehicles, which can produce significant side effects.

In another example, various compounds – including neuropeptides such as enkephalins, the *N*-methyl-D-aspartate receptor antagonist MRZ 2/576, and the chemotherapeutic drug doxorubicin – have been attached to the surface of poly(butylcyanoacrylate) nanoparticles coated with polysorbate 80 [3-7]. The polysorbate on the surface of the nanoparticles adsorb apolipoproteins B and E and are taken up by brain capillary endothelial cells via receptor-mediated endocytosis. Nanoparticle-mediated delivery of doxorubicin is being explored in a rodent model of glioblastoma [3,8]. Importantly, recent work in a rat glioblastoma model revealed significant remission with minimal toxicity, setting the stage for potential clinical trials [8].

The delivery of other drugs is also being investigated. Dalargin is a hexapeptide analog of leucine-enkephalin containing D-alanine, which produces CNS analgesia when it is delivered intracerebroventricularly, but it has no analgesic effects if it is administered systemically, specifically because it cannot cross the BBB on its own [9,10]. [3H]Dalargin was conjugated to the same poly(butylcyanoacrylate) nanoparticles described above, injected systemically into mice, and demonstrated by radiolabeling to cross the BBB and accumulate in brain [10]. Other, similar studies have also demonstrated delivery of dalargin using polysorbate 80-coated nanoparticles [11]. Other polysorbate 80 nanoparticles have been chemically conjugated to the hydrophilic drug diminazenediaceturate (diminazene) and proposed as a novel treatment approach for second stage African trypanosomiasis [12]. In other work, PEG-treated polyalkylcyanoacrylate nanoparticles were shown to cross the BBB and accumulate at high densities in the brain in experimental autoimmune encephalomyelitis [13], a model of multiple sclerosis [14,15].

For other applications, molecules other than drugs must cross the BBB for therapeutic or diagnostic reasons, including oligonucleotides, genes, and contrast agents. Solid lipid nanoparticles consisting of microemulsions of solidified oil nanodroplets loaded with iron oxide and injected systemically into rats have been shown to cross the BBB and accumulate in the brain with long-lasting kinetics [16]. Iron oxides are classic superparamagnetic magnetic resonance imaging (MRI) contrast agents. Because iron oxides are insoluble in water, they must be delivered as modified colloids for clinical applications, which is usually achieved by coating them with hydrophilic molecules, such as dextrans [17]. Therefore, the delivery vehicle used is critical in determining the functional properties of the contrast agent. By taking advantage of the ability of these solid lipid nanoparticles to cross the BBB, nanoparticles complexed with iron oxides may provide new ways to image the CNS using MRI.

Other work has focused on the delivery of oligonucleotides in an *in vivo *mouse model and an *in vitro *endothelial cell model, with the aim being to develop novel treatments for neurodegenerative disorders [18]. The investigators synthesized a nanogel consisting of cross-linked PEG and polyethylenimine that spontaneously encapsulated negatively charged oligonucleotides. In their *in vivo *model, they demonstrated that intravenous injections resulted in a 15-fold accumulation of oligonucleotides in the brain after 1 hour, with a concurrent twofold decrease in accumulation in liver and spleen when compared with freely administered oligonucleotides (not encapsulated in nanogel particles).

A related area is the delivery of genes to the CNS for gene therapy. A tyrosine hydroxylase (TH) expression plasmid was delivered to the striatum of adult rats using PEG immunoliposome nanoparticles in order to normalize TH expression levels in the 6-hydroxydopamine rat model of Parkinson's disease [19]. Using specific antibodies to transferrin receptors conjugated to the nanoparticles, TH plasmids were shown to be expressed throughout the striatum.

## Conclusion

Nanotechnology-based approaches to targeted delivery of drugs and other compounds across the BBB may potentially be engineered to carry out specific functions as needed. The drug itself – in other words the biologically active component being delivered, whatever that may be – constitutes one element of a nanoengineered complex. The rest of the complex is designed to carry out other key functions, including shielding the active drug from producing systemic side effects, being prematurely cleared or metabolized, crossing the BBB, and targeting specific cells after it has gained access to the CNS. Implicitly, all of this must be achieved by any drug intended to have CNS effects, regardless of whether it is part of a nanoengineered complex.

An important advantage of a nanotechnological approach, as compared with the administration of free drug or the drug associated with a nonfunctional vehicle, is that these critical requirements do not need to be carried out by the active compound, but by supporting parts of the engineered complex. This allows the design of the active drug to be tailored for maximal efficacy. Currently, most nanoengineered systems for crossing the BBB take advantage of drugs that are already in clinical use and therefore have greater potential for reaching the clinic relatively quickly.

In addition to the delivery of drugs and other compounds across the BBB for therapeutic purposes, the ability to cross the BBB selectively and efficiently in animal models using nanoengineered technologies will have a significant impact on research that focuses on the normal physiology of the CNS and its pathology, by allowing targeted *in vivo *studies of specific cells and processes using methods that take advantage of the intact live organism. Ideally, methods for crossing the BBB will complement other nanotechnological tools being developed to study the CNS, including quantum dot labeling and imaging [20].

## List of abbreviations used

BBB: blood-brain barrier; CNS: central nervous system; PEG: polyethylene glycol.

## Competing interests

The author declares that they have no competing interests.
